# AZD1775 sensitizes T cell acute lymphoblastic leukemia cells to cytarabine by promoting apoptosis over DNA repair

**DOI:** 10.18632/oncotarget.4830

**Published:** 2015-08-10

**Authors:** James B. Ford, Dmitry Baturin, Tamara M. Burleson, Annemie A. Van Linden, Yong-Mi Kim, Christopher C. Porter

**Affiliations:** ^1^ Department of Pediatrics, University of Colorado School of Medicine, Aurora, Colorado, USA; ^2^ University of Nebraska Medical Center, Omaha, Nebraska, USA; ^3^ Medical Scientist Training Program, University of Colorado School of Medicine, Aurora, Colorado, USA; ^4^ Department of Pediatrics, University of Southern California, Los Angeles, California, USA

**Keywords:** Wee1, DNA damage, leukemia, experimental therapeutics, kinase

## Abstract

While some children with acute lymphoblastic leukemia (ALL) have excellent prognoses, the prognosis for adults and children with T cell ALL is more guarded. Treatment for T-ALL is heavily dependent upon antimetabolite chemotherapeutics, including cytarabine. Targeted inhibition of WEE1 with AZD1775 has emerged as a strategy to sensitize cancer cells to cytarabine and other chemotherapeutics. We sought to determine if this strategy would be effective for T-ALL with clinically relevant anti-leukemia agents. We found that AZD1775 sensitizes T-ALL cells to several traditional anti-leukemia agents, acting synergistically with cytarabine by enhancing DNA damage and apoptosis. In addition to increased phosphorylation of H2AX at serine 139 (γH2AX), AZD1775 led to increased phosphorylation of H2AX at tyrosine 142, a signaling event associated with promotion of apoptosis over DNA repair. In a xenograft model of T-ALL, the addition of AZD1775 to cytarabine slowed leukemia progression and prolonged survival. Inhibition of WEE1 with AZD1775 sensitizes T-ALL to several anti-leukemia agents, particularly cytarabine. Mechanistically, AZD1775 promotes apoptosis over DNA repair in cells treated with cytarabine. These data support the development of clinical trials including AZD1775 in combination with conventional chemotherapy for acute leukemia.

## INTRODUCTION

T cell acute lymphoblastic leukemia (T-ALL) accounts for around 20% of ALL cases and has historically been a high-risk leukemia in both pediatric and adult populations [1, 2]. Patients with T-ALL often present with high-risk features, such as high white blood cell counts and infiltration of the central nervous system. While overall survival rates in patients with T-ALL have improved with advances in risk-adapted chemotherapy regimens, in children early relapses occur more often in those with T-ALL as compared to B-ALL, leading to a lower comparative overall survival [3, 4]. Chemotherapy for T-ALL is heavily reliant on antimetabolites, such as cytarabine and methotrexate [5, 6], among other cytotoxic chemotherapeutics, which lead to acute as well as long term toxicities. Novel therapeutic strategies are needed for T-ALL and other high-risk leukemias to improve survival and decrease exposure to cytotoxic chemotherapy.

One such strategy would be to pharmacologically sensitize leukemia cells to standard of care anti-leukemia agents. Several cell cycle checkpoint proteins have been studied as chemosensitizing targets for cancer therapy including ATR, CHK1 and WEE1. WEE1 is a protein kinase that acts as a cell cycle checkpoint protein by inhibiting CDK1 and CDK2 (and, hence cell cycle progression) by phosphorylation at Tyr15 [7, 8]. WEE1 is active during normal S phase, or in the face of DNA damage, to ensure accurate replication of the genome prior to mitosis. We, and others, identified WEE1 as a critical mediator of acute myeloid leukemia (AML) cell survival after exposure to cytarabine [9, 10]. Inhibition of WEE1 abrogates the S phase arrest and enhances the apoptosis induced by cytarabine in AML cells, independent of p53 functionality [9, 11]. Treatment of mice with cytarabine and AZD1775 (formerly MK-1775), a specific WEE1 inhibitor [12], slows the progression of murine AML and prolongs survival more than cytarabine alone [11]. However, WEE1 inhibition has not been well studied as a target for acute lymphoblastic leukemia, nor in combination with other, commonly used, conventional chemotherapeutics for ALL.

While AZD1775 is currently in phase II trials for some solid tumors, there are currently no approved trials of AZD1775 for patients with leukemia. We sought to determine whether AZD1775 sensitizes T cell ALL to a wide variety of clinically relevant anti-leukemia agents. We found that AZD1775 synergistically inhibits T-ALL cells treated with cytarabine, but not several other conventional agents, including doxorubicin and etoposide. WEE1 inhibition also impairs the proliferative capacity of T-ALL cells after exposure to cytarabine or 6-thioguanine (6TG), and enhances the apoptosis induced by these drugs. The combinatorial effect of AZD1775 and cytarabine appears to be due in part due to enhanced DNA damage, but we also observed increased phosphorylation of H2AX at tyrosine 142 in combination treated cells as compared to either drug alone. As this phosphorylation site has been associated with a cellular decision between DNA repair and survival versus apoptosis [13], these data suggest that WEE1 may function to promote survival in the context of DNA damage, in addition to its role in stalling cell cycle progression. We also treated mice with human T-ALL with cytarabine and/or AZD1775 and found that we could detect a reduction in phosphorylation of CDK1/2 in AZD1775 treated mice, and that AZD1775 in combination with cytarabine reduced disease burden and prolonged survival more than either drug alone. These data support the development of clinical trials testing AZD1775 in combination with conventional chemotherapeutics for the treatment of acute leukemia.

## RESULTS

**Figure 1:**
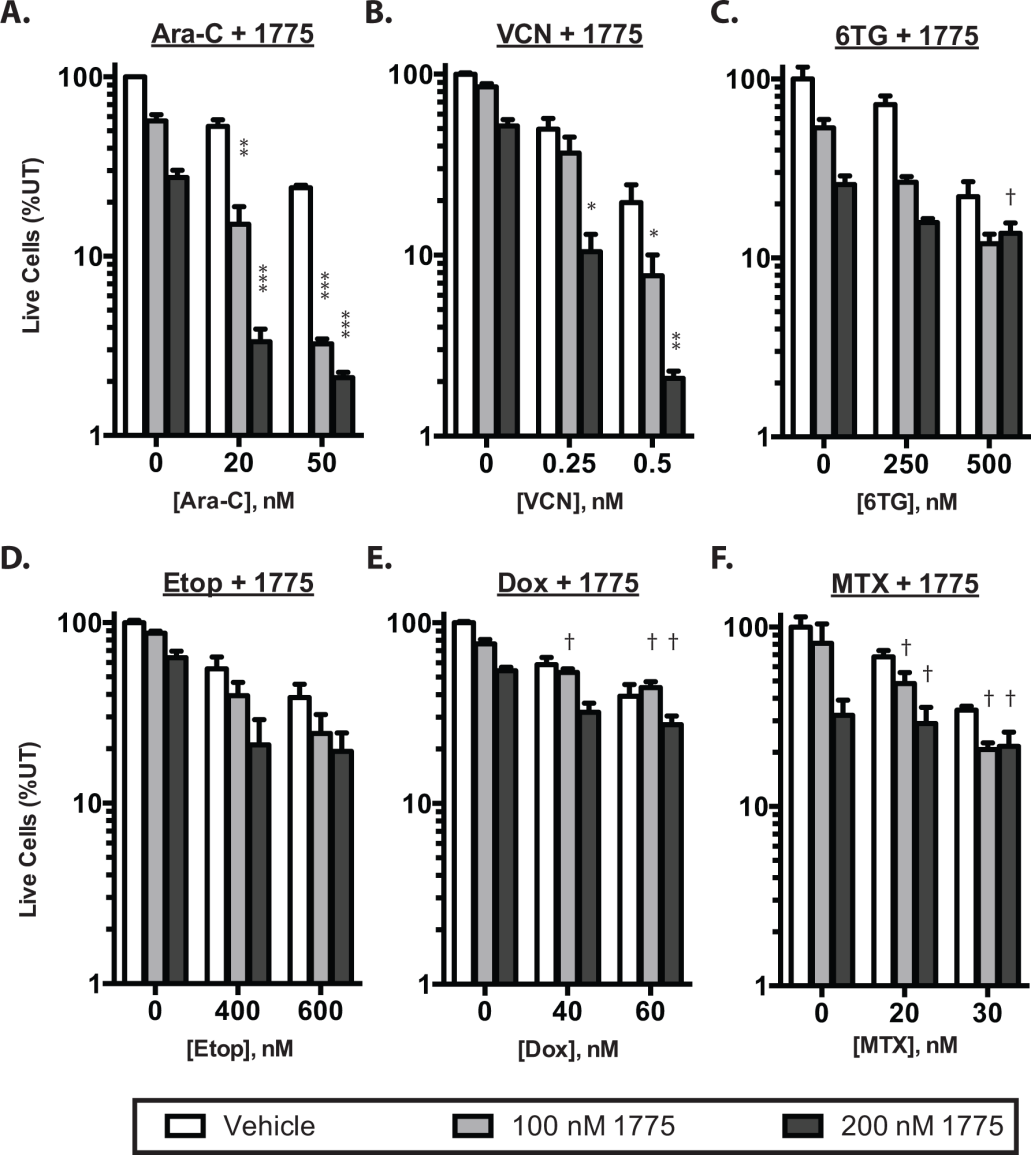
AZD1775 synergistically enhances the effect of cytarabine Jurkat cells were cultured for 72 hours and then counted by propidium iodide exclusion and flow cytometry. The number of live cells is depicted as a percentage of untreated controls. Cells were treated with AZD1775 0, 100 or 200 nM in combinataion with: **A.** cytarabine (Ara-C) **B.** vincristine (VCN) **C.** 6-thioguanine (6TG), **D.** etoposide (Etop), **E.** doxorubicin (Dox) or **F.** Methotrexate (MTX) at the indicated concentrations. Combination index (CI) values were calculated and synergistic inhibition is indicated by asterisks (*CI < 0.8; **CI < 0.6; ***CI < 0.4) whereas antagonism is indicated by a dagger (†CI > 1.2).

**Figure 2:**
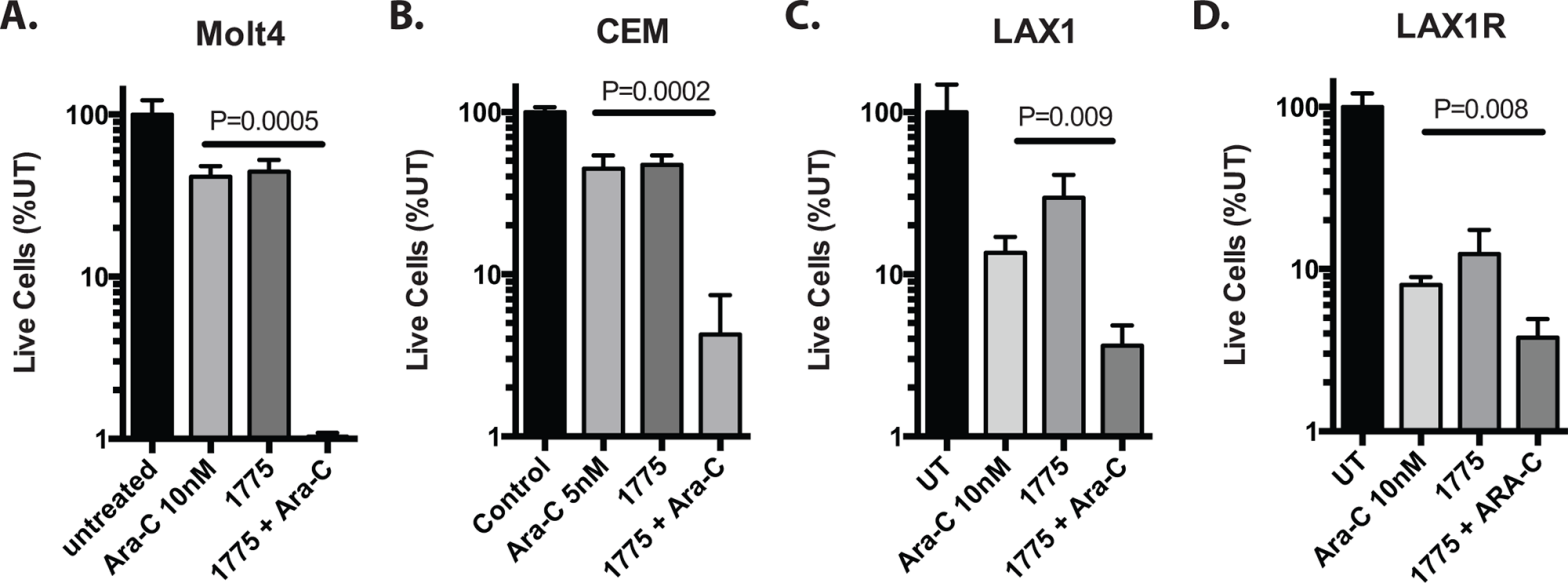
AZD1775 sensitizes T-ALL cell lines and patient derived samples to cytarabine Cells were cultured with the cytarabine at the indicated concentrations with or without AZD1775 (200 nM). Live cell numbers are depicted as a percentage of untreated. **A.** Molt4 and **B.** CEM cells were counted at 72 hours. **C.** LAX1 and **D.** LAX1R cells are derived from a patient with T-ALL at initial diagnosis and relapse respectively. Due to long doubling times, these cells were counted and replated with fresh drug after 6 days, cultured another 6 days, and then counted again. Extrapolated cell counts relative to untreated are depicted.

To determine whether inhibition of WEE1 sensitizes ALL cell lines to clinically relevant chemotherapeutics, we first treated Jurkat cells with a panel of clinically relevant anti-leukemia agents with a range of doses to estimate the dose that inhibited proliferation by 50% (IC_50;_ Supplementary Figure S1A, S1B). We then treated cells with and without AZD1775 at a subset of doses and combinations (Figure 1 and Supplementary Tables S1–S6). We observed significant reduction in the number of live cells after 72 hours of culture in the presence of AZD-1775 with cytarabine and vincristine (Figure 1A, 1B), and calculation of the combination index [14] suggested synergistic activity between AZD1775 and cytarabine or vincristine, albeit with limited numbers of combinations. The combinatorial effect was not synergistic at this time point with 6TG and etoposide, and was antagonistic at some doses of 6-thiguanine, doxorubicin and methotrexate (Figure 1C, 1D, 1E, 1F). Evaluation for synergy using larger numbers of combinations using 3-dimensional modeling [15] indicated that at this time point, cytarabine is the only of these drugs with which AZD1775 is synergistic (Supplementary Figure S1C). No chemosensitization was seen with asparaginase or methylprednisolone (not shown). We saw similar chemosensitization to cytarabine in two additional T-ALL cell lines, Molt4 and CEM (Figure 2A, 2B), as well as samples derived from a patient at the time of initial diagnosis and relapse (Figure 2C, 2D). Importantly, the relapse sample remained sensitive to the combination of cytarabine and AZD-1775, suggesting that such a strategy may be useful in the relapse setting.

**Figure 3:**
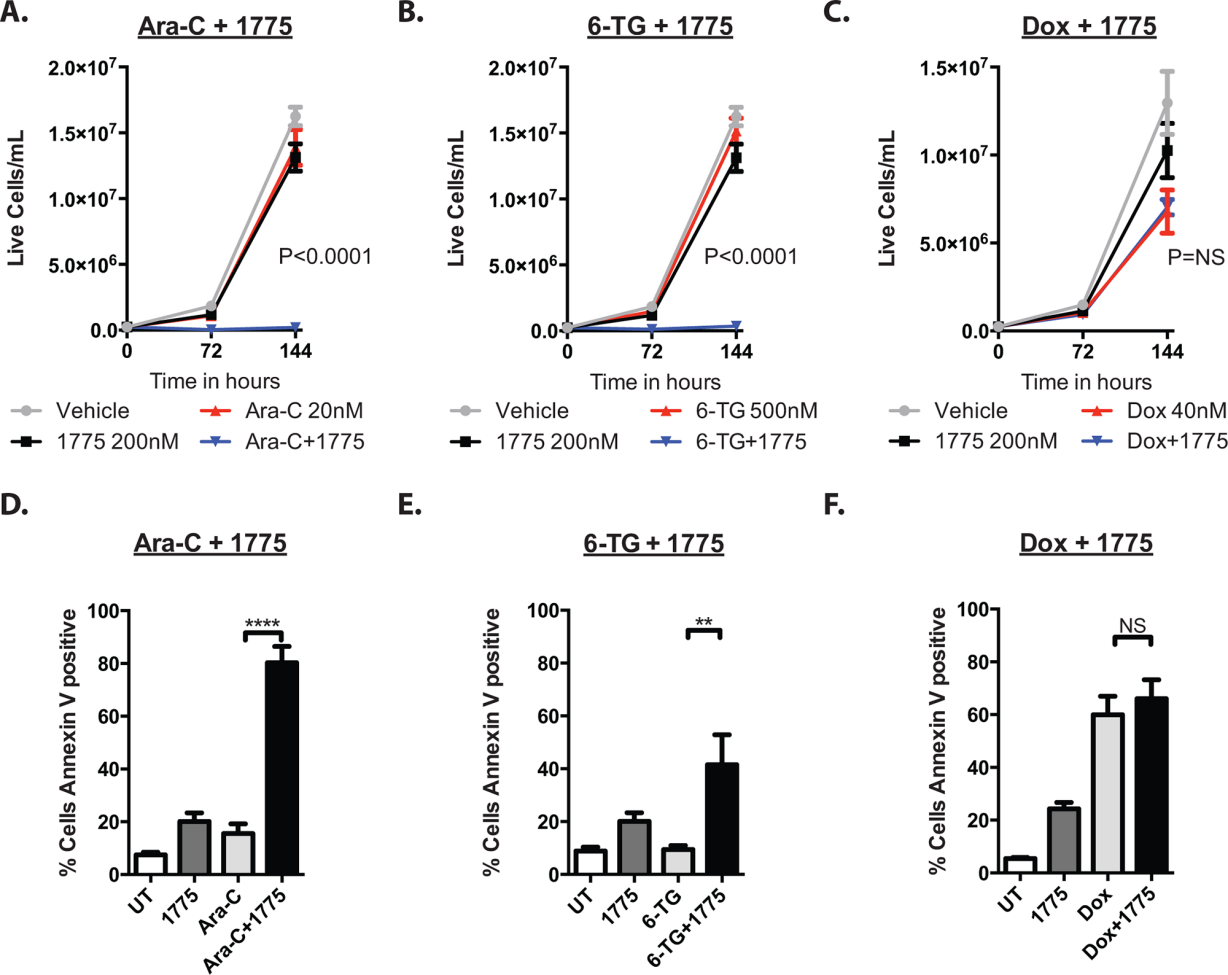
AZD1775 impairs the proliferative capacity and enhances apoptosis of leukemia cells after exposure to cytarabine and 6-thioguanine, but not doxorubicin **A, B, C.** Jurkat cells were cultured with AZD1775 200 nM with or without ARA-C (20 nM), 6TG (500 nm) or Doxorubicin (40 nM) for 72 hours, counted and were split 1:10 into fresh media, and cultured another 72 hours without treatment and counted again. Extrapolated cell concentrations are depicted. *P* values represent Student's *t* test comparing chemotherapy to chemotherapy plus AZD1775. **D, E, F.** Jurkat cells were cultured with AZD1775 with or without ARA-C, 6-TG or doxorubicin as in A, B, C for 72 hours, stained for Annexin V, and analyzed by flow cytometry. **P < 0.01; ****P < 0.0001.

As cancer cells often regain proliferative capacity after treatment with conventional chemotherapeutics, we sought to determine if AZD1775 would prevent proliferation after treatment. Thus, cells were cultured with AZD1775 with or without cytarabine, 6-thioguanine or doxorubicin for 72 hours and then re-plated without drug and cultured another 72 hours. In these experiments, the cells recovered after treatment with the chemotherapy alone, but did not recover after treatment with AZD1775 and cytarabine or 6-thioguanine (Figure 3A, 3B). Interestingly, the leukemia cells were able to recover after treatment with AZD1775 and doxorubicin (Figure 3C), suggesting that WEE1 inhibition may be most toxic with anti-metabolite chemotherapeutics.

**Figure 4:**
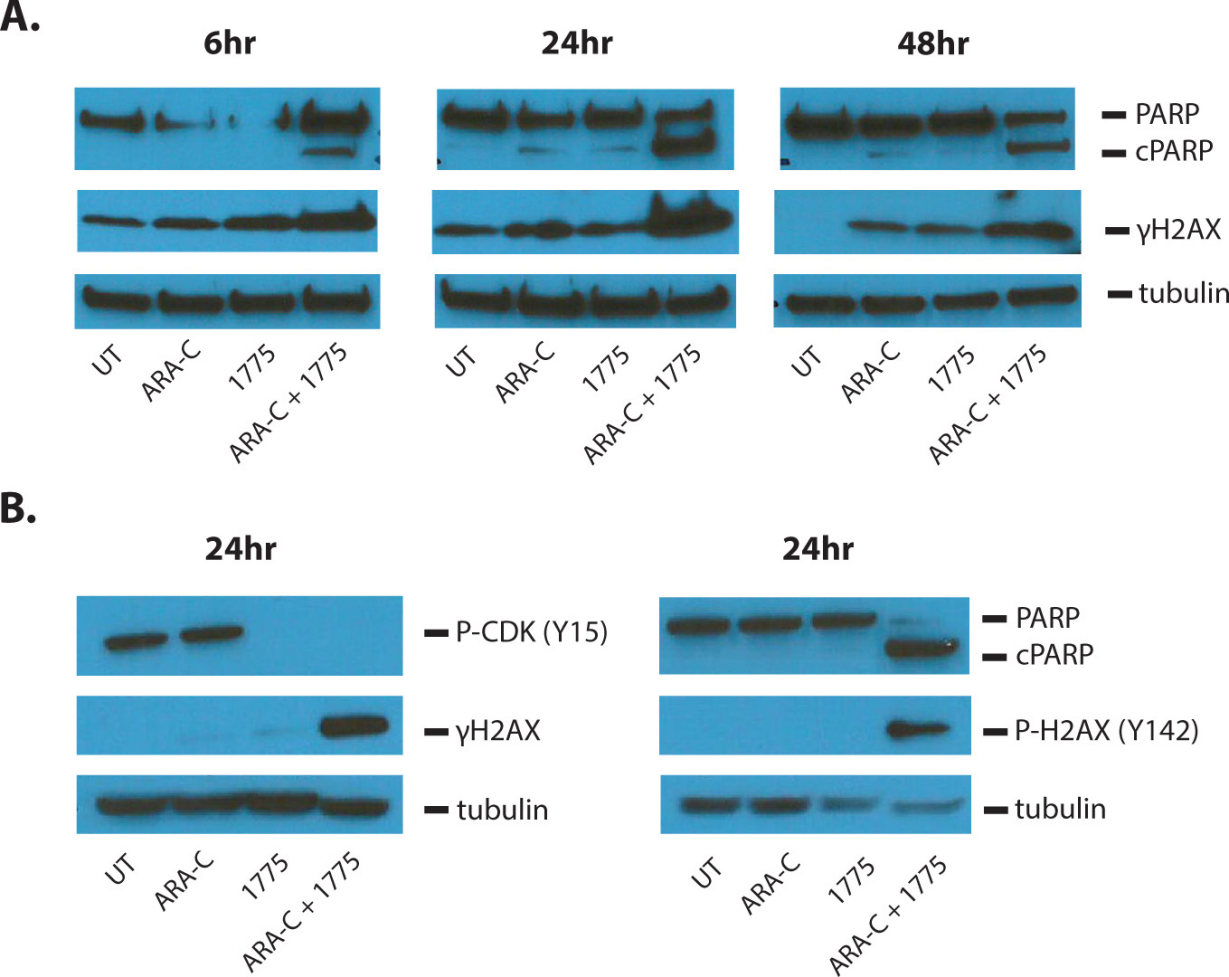
AZD1775 enhances DNA damage and apoptosis induced by cytarabine Jurkat cells were treated for the indicated amount of time with cytarabine 50 nM and/or AZD1775 200 nM. **A.** Protein lysates were subjected to western blot with antibodies for PARP (which also detects cleaved PARP (cPARP)), phosphorylated H2AX-S139 (γH2AX), and tubulin. **B.** Protein lysates were subjected to western blot for phosphorylated CDK-Y15, γH2AX, tubulin, PARP and phosphorylated H2AX-Y142.

We next sought to determine whether the addition of AZD1775 to these agents induced more apoptosis than the chemotherapy alone. Indeed, at 72 hours, the addition of the WEE1 inhibitor to cytarabine and 6-thioguanine led to significantly more apoptosis than with either agent alone, as measured by Annexin V staining (Figure 3D, 3E). However, we did not see an increase in apoptosis when AZD1775 was combined with doxorubicin (Figure 3F). Consistent with enhanced apoptosis due to AZD1775 and cytarabine treatment, at 6 hours of treatment we could detect higher levels of cleaved PARP that increased by 24 hours, when compared to those treated with either drug alone (Figure 4A). We also detected more phosphorylation of H2AX at serine 139 (γH2AX), indicative of DNA damage. In addition, we observed enhanced phosphorylation of H2AX at tyrosine 142 (Figure 4B), which is associated with a cellular decision to promote apoptosis over DNA repair and survival [13].

**Figure 5:**
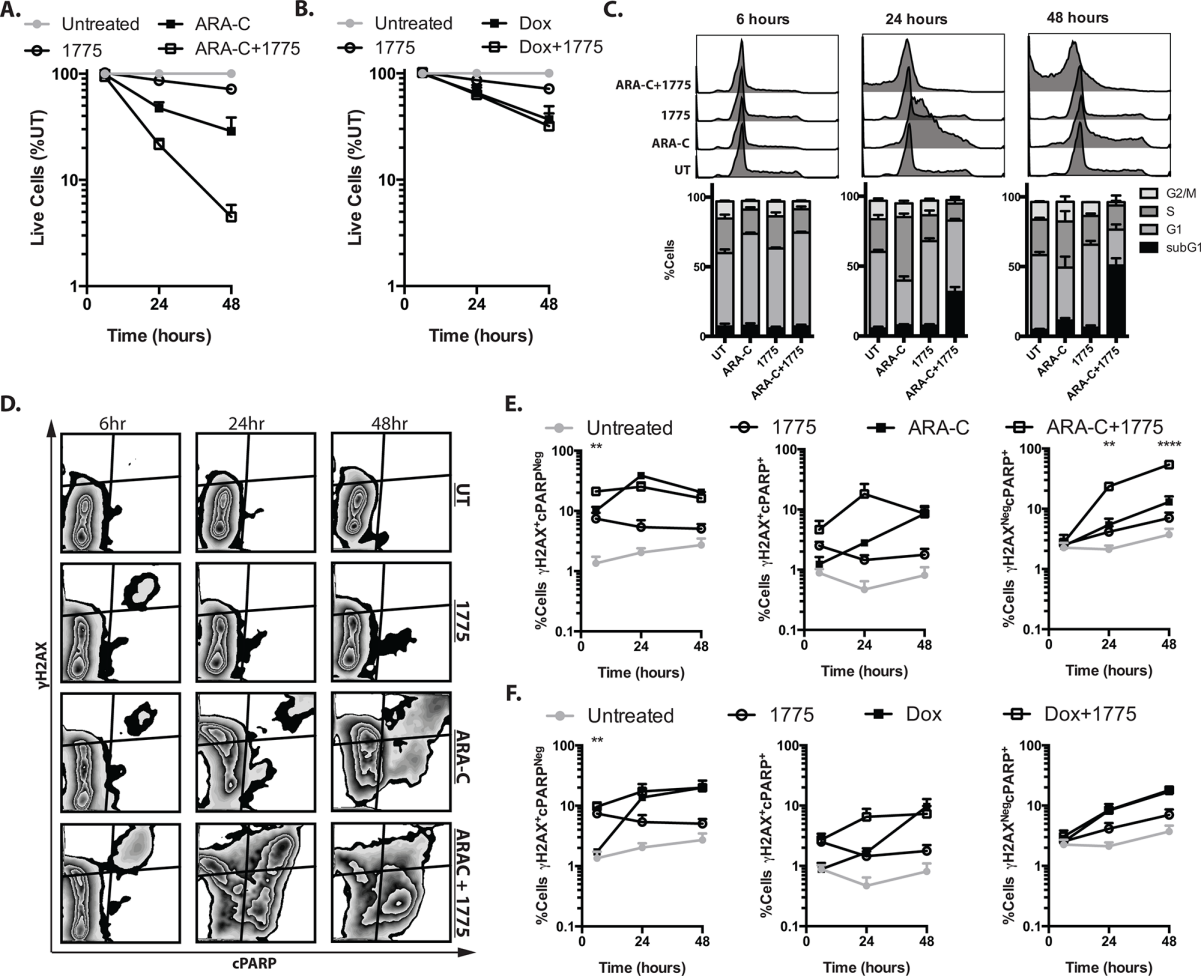
AZD1775 promotes DNA damage and apoptosis with cytarabine but not doxorubicin Jurkat cells were treated with cytarabine 50 nM, doxorubicin, 50 nM, AZD1775 200 nM or in combination as indicated, and harvested at 6, 24 and 48 hours. **A, B.** Cells were counted by propidium iodide exclusion and flow cytometry. The number of live cells, relative to untreated is depicted. **C.** Cells were fixed and stained with 7-AAD to assess cell cycle distribution by DNA content. **D.** Fixed and permeabolized cells were stained with fluorescently tagged antibodies directed against γH2AX and cleaved PARP (cPARP). **E, F.** The percentages of γH2AX^+^/cPARP^neg^, γH2AX^+^/cPARP^+^ and γH2AX^neg^/cPARP^+^ cells is depicted.

In order to better understand the mechanisms of combinatorial cytotoxicity with AZD1775 and cytarabine, we first analyzed cell numbers and cell cycle progression at earlier time points than previously examined, presuming that the observations at 72 hours represent the culmination of processes evolving over that time. Indeed, after 24 hrs we observed enhanced reduction in cell numbers in cells treated with AZD1775 and cytarabine compared to those treated with cytarabine alone (*P* = 0.01). This enhanced effect was not seen with AZD1775 in combination with doxorubicin (Figure 5A, 5B). At this same time point we also saw an abrogation of the cytarabine-induced S phase arrest, as well as the emergence of a sub G1 population, when cells were also treated with AZD1775 (Figure 5C). By 48 hours, cytarabine treated cells appeared to have overcome their S phase arrest, however the combination treated cells exhibited greater percentage of sub G1 cells, suggesting extensive apoptosis. While there was some abrogation of cell cycle changes due to doxorubicin treatment, the addition of AZD1775 did not enhance the subG1 population in combination with doxorubicin (Supplementary Figure S2). To analyze DNA damage and apoptosis at the single cell level, we performed flow cytometry for γH2AX and cleaved PARP (Figure 5D, 5E, 5F and Supplementary Figure S2). We found that as early as 6 hours, there is a significant increase in the percentage of γH2AX^+^/cPARP^neg^ cells in the AZD1775 and cytarabine treated cells as compared to cytarabine alone. By 24 hours, there was a significant increase in the percentage of γH2AX^neg^/cPARP^+^ cells that increased further at 48 hours. These findings suggest that inhibition of WEE1 influences the DNA damage response resulting in both a reduction of γH2AX that is necessary for DNA damage repair, and the promotion of apoptosis. This is consistent with the finding of enhanced phosphorylation of H2AX at tyrosine 142. Interestingly, we did not see a similar difference in staining of γH2AX and cPARP staining when AZD1775 was added to doxorubicin (Supplementary Figure S2), again suggesting a contextual benefit of WEE1 inhibition.

**Figure 6:**
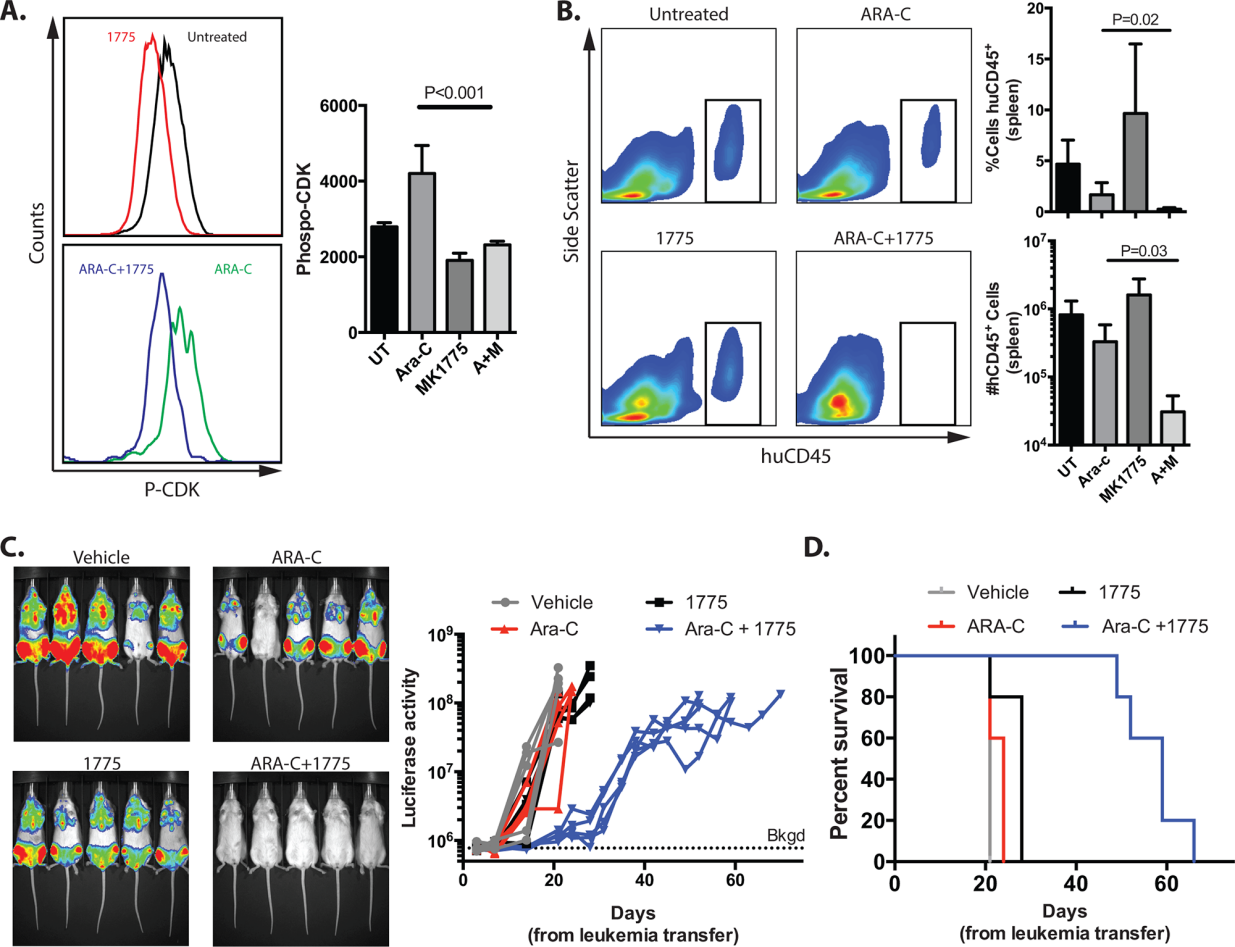
AZD1775 inhibits WEE1 in human leukemia cells *in vivo* and enhances the efficacy of cytarabine in mice with human leukemia Luciferase tagged Jurkat cells were injected into sub-lethally irradiated NRG mice that were treated with cytarabine (ARA-C), AZD1775, both or vehicles. Mice were imaged periodically by IVIS for luciferase activity. Upon euthanasia, spleens were analyzed by flow cytometry. **A.** After 5 days of therapy, recipients were euthanized, and splenic cells were stained with antibodies against human CD45 (huCD45), fixed and permeabolized, and stained for phosphorylated CDK-Y15. Representative histograms are shown. The median fluorescence intensity of P-CDK staining in human cells from is shown (*n* = 5/group). **B.** Representative histograms of huCD45 staining from cells harvested from spleens. The percentage and total numbers of huCD45 cells are depicted graphically. **C.** Representative IVIS imaging at day 14 and signal from individual mice over time. The background (Bkgd) level of signal from mice without leukemia averaged 7.75 × 10^5^ photons/sec. Mice were euthanized when moribund or the luciferase activity reached 1 × 10^8^ photons/sec. **D.** Kaplan-Meier curve of mice with human leukemia treated as indicated. Data are representative of 2 independent experiments with similar results.

To determine whether the addition of AZD1775 to cytarabine may be a tolerable and effective combination *in vivo*, we modeled human leukemia in immune-compromised mice. We first demonstrated that AZD1775 effectively inhibits WEE1 kinase activity *in vivo* at the dose administered by performing flow cytometry for phospho-CDK in human leukemia cells harvested from mice (Figure 6A). Cytarabine alone increased the mean fluorescence intensity of phospo-CDK staining, which was abrogated by treatment with AZD1775. Consistent with findings *in vitro*, we observed a greater reduction in the percentage and number of human leukemia cells in the spleens of mice after treatment with AZD1775 and cytarabine, as compared to cytarabine alone (Figure 6B). In a longer-term experiment in which leukemia burden was measured non-invasively with IVIS imaging, the combination of AZD1775 and cytarabine significantly slowed disease progression (*P* = 0.01 at day 21) and prolonged survival (*P* = 0.003), as compared to cytarabine alone (Figure 6C, 6D).

## DISCUSSION

Novel therapeutic strategies are needed for high-risk leukemias, including T-ALL. In this report, we demonstrate that AZD1775 sensitizes multiple T-ALL cell lines to some, but not all, conventional chemotherapeutics commonly used to treat ALL. We investigated the mechanism of synergistic inhibition with AZD1775 and cytarabine, and found that AZD1775 abrogates the S phase arrest and enhances the DNA damage and apoptosis induced by cytarabine. Lastly, we demonstrated that AZD1775 inhibits WEE1 function in leukemia cells *in vivo*, delays leukemia progression and prolongs survival better than cytarabine alone.

Several agents targeting cell cycle checkpoints as a way to augment the treatment of leukemia have been investigated. Antimetabolite chemotherapeutics, nucleoside analogues in particular, result in activation of the ATR-CHK1-CDC25/WEE1 signaling cascade, stalling cell cycle progression and allowing for DNA damage repair prior to mitosis [16, 17]. Due to its central and relatively non-redundant role in the DNA damage response, the study and development of CHK1 inhibitors is the most advanced. UCN-01, a CHK1 inhibitor with a number of other kinase targets, abrogates the S phase arrest and survival of acute myeloid leukemia cells in preclinical studies [18, 19], but has unfavorable pharmacokinetics and toxicity that precluded further development. More recently, a specific CHK1 inhibitor was tested in combination with cytarabine in a Phase I trial in patients with refractory acute leukemias, with favorable pharmacodynamics and safety [20]. Specific ATR inhibitors are in development (http://clinicaltrials.gov), but the results of Phase 1 trials have yet to be published. AZD1775 is a specific WEE1 inhibitor that was well tolerated as a single agent, not reaching a maximum tolerated dose in a dose finding study [21], and is currently being tested in combination with several different agents for solid tumors (http://clinicaltrials.gov). Our data builds on mounting evidence that WEE1 inhibition may augment the effect of chemotherapy for the treatment of leukemias [9, 10, 22, 23].

With this report we provide several important findings in support of clinical trials testing AZD1775 for the treatment of leukemia in combination with chemotherapy. To our knowledge, this is the first demonstration of the efficacy of WEE1 inhibition with cytarabine in the treatment of human leukemia *in vivo*. While we have shown a benefit in combining AZD1775 with cytarabine for acute myeloid leukemia *in vivo* [11] these experiments were done with a murine leukemia. Both of our studies demonstrate that AZD1775 can be combined with cytarabine without overt toxicity at these doses.

Importantly, we demonstrated the efficacy of AZD1775 in combination with cytarabine in multiple human T-ALL cell lines, as well as in human derived samples from the time of diagnosis and also at relapse from the same patient. The fact that the relapse sample demonstrated chemosensitization to cytarabine suggests that previous exposure to multi-agent chemotherapy may not abrogate the benefit of AZD1775 for those in whom it is likely to be tested first. We also tested multiple clinically relevant anti-leukemia agents with AZD1775 and, importantly, found chemosensitization only with cytarabine or vincristine when measured after 72 hours of treatment. While we were able to calculate that the interaction between AZD1775 and cytarabine was synergistic, it is important to acknowledge that the determination of synergistic drug interactions in cell lines has limitations and does not necessarily predict *in vivo* activity. However, our *in vivo* data presented here and previously [11], suggest that leukemia-cell sensitization to cytarabine using AZD1775 can be achieved *in vivo*.

Here we were also able to demonstrate that phosphorylated CDK (Y15) in human leukemia cells *in vivo* can be used as a biomarker of target engagement for AZD1775. This may be a particularly useful, relatively non-invasive, means of assessing cancer-cell specific pharmacodynamics in early phase clinical trials for patients with leukemia, as circulating peripheral blasts may be sampled from the peripheral blood for flow cytometric analysis.

Lastly, we demonstrate that inhibition of WEE1 dramatically alters the response of T-ALL cells to cytarabine by abrogating S phase arrest and enhancing DNA damage induced by cytarabine, and by promoting apoptosis over DNA damage repair, as evidenced by phosphorylation of H2AX at tyrosine 142. The latter finding is novel, and raises the possibility that WEE1 has an underappreciated function in promoting DNA damage repair and survival. This hypothesis is supported by a recent report demonstrating that WEE1 inhibition impairs homologous recombination due to inhibitory phosphorylation of BRCA2 by CDK1 [24]. Whether WEE1 plays a distinct role in promoting survival independent of its function in DNA repair remains to be explored.

In summary, this report demonstrates the chemosensitizing effect of AZD1775, highlighting cytarabine in particular, with which it may be most effectively combined. Importantly, we show for the first time that AZD1775 can be combined with cytarabine effectively to treat human leukemia *in vivo*. In addition, we provide more understanding about the mechanism of chemosensitization with cytarabine, showing that AZD1775 promotes apoptosis over DNA repair and survival. These data and others support clinical trials testing AZD1775 in combination with chemotherapy for acute leukemia.

## MATERIALS AND METHODS

### Cell lines and tissue culture

Jurkat, Molt4 and CEM cell lines were generous gifts form the laboratory of Dr. Douglas Graham. Cell lines were tested for mycoplasma and DNA fingerprinted utilizing STR DNA fingerprinting (Life Technologies), as previously described, and stock vials were subsequently stored in liquid nitrogen. Patient derived samples, LAX1 and LAX1R, were obtained at initial diagnosis and relapse, respectively, from a patient with T-ALL after informed consent was obtained, and in compliance with the Institutional Review Board of the University of Southern California. Cells were cultured at 37°C in humidified air supplemented with 5% CO_2_ in RPMI supplemented with 10% FBS and antibiotics, except for the LAX1 and LAX1R that were cultured in αMEM with 20% FBS, 2.5% HEPES and antibiotics. Cells were seeded at 2.5 × 10^5^ cells/mL for experimentation and were counted at the indicated time points by propidium iodide (Sigma-Aldrich) exclusion and flow cytometry (Guava EasyCyte Plus, Millipore).

### Chemotherapy, antibodies and reagents

AZD1775 was provided by the National Cancer Institute (Bethesda, MD) and Merck Sharp & Dohme Corporation. Conventional chemotherapeutic agents were purchased from Sigma-Aldrich. Antibodies against phosphorylated CDK1 (Y15) were purchased from Cell Signaling Technology; phosphorylated H2AX (Y142) from Abcam; cPARP, and γH2AX from BD Biosciences, and PE-linked human CD45 from eBioscience.

### Flow cytometry

Apoptosis was measured with the Guava EasyCytePlus using Nexin reagent per the manufacturer's protocol (Millipore). Cell cycle, DNA damage and apoptosis were measured simultaneously using the Apoptosis, DNA damage and Cell Proliferation Kit (BD Biosciences) according to the manufacturer's instructions. Fixation and permeabilization buffers from this kit were used to prepare cells for staining with PE-linked human CD45 and phopho-CDK1 antibodies followed by secondary staining with anti-rabbit Alexa Fluor 488. Stained cells were analyzed on a Gallios 561 flow cytometer (Beckman Coulter).

### Animal experiments

Female NOD.Cg-Rag1*^tm1Mom^* Il2rg*^tm1Wjl^*/SzJ (NRG) mice, 6–8 weeks old, were purchased from Jackson Laboratories. All of the mice were housed in sterile micro-isolators at the Center for Comparative Medicine at the University of Colorado Anschutz Medical Campus (AMC) and remained in quarantine for 1 week prior to initiation of the experiments. Mice were sublethally irradiated with 175 cGy, then injected with 5 × 10^6^ luciferase expressing Jurkat cells [25]. Mice were treated with cytarabine 5 mg/kg/dose via intraperitoneal injection and/or with AZD1775 40 mg/kg twice a day via oral gavage. Control mice were given an intraperitoneal injection with phosphate buffered solution, and had twice daily oral gavage treatments with the methylcellulose vehicle of the AZD1775. Mice were treated 5 days per week. Luciferase activity was measured 5 minutes after injection of luciferin using an IVIS2000 imaging system (Xenogen). All of the animal studies were approved by the Institutional Animal Care and Use Committee of the University of Colorado AMC.

### Data analysis

Graphpad Prism 5 and Excel were used for data analysis and graphing. Graphs depict the mean from replicate experiments and error bars portray the standard error of the mean. Combination Index values were calculated using the method of Chou and Talalay [14] with CalcuSyn (Biosoft). Three-dimensional modeling of combination drug effects and estimation of synergy were calculated using MacSynergy II [15]. Student's *t* test was used to detect significant differences between 2 samples. The Mantel-Cox (log-rank) test was used to test for significant differences in survival. Except for *in vivo* studies, all experiments were completed in duplicate or triplicate and were repeated at least 3 times, unless explicitly stated.

## SUPPLEMENTARY FIGURES AND TABLES



## References

[1] Aifantis I, Raetz E, Buonamici S (2008). Molecular pathogenesis of T-cell leukaemia and lymphoma. Nature reviews Immunology.

[2] Van Vlierberghe P, Ferrando A (2012). The molecular basis of T cell acute lymphoblastic leukemia. J Clin Invest.

[3] Hunger SP, Lu X, Devidas M, Camitta BM, Gaynon PS, Winick NJ, Reaman GH, Carroll WL (2012). Improved survival for children and adolescents with acute lymphoblastic leukemia between 1990 and 2005: a report from the children's oncology group. Journal of clinical oncology : official journal of the American Society of Clinical Oncology.

[4] Goldberg JM, Silverman LB, Levy DE, Dalton VK, Gelber RD, Lehmann L, Cohen HJ, Sallan SE, Asselin BL (2003). Childhood T-cell acute lymphoblastic leukemia: the Dana-Farber Cancer Institute acute lymphoblastic leukemia consortium experience. Journal of clinical oncology : official journal of the American Society of Clinical Oncology.

[5] Winter SS, Holdsworth MT, Devidas M, Raisch DW, Chauvenet A, Ravindranath Y, Ducore JM, Amylon MD (2006). Antimetabolite-based therapy in childhood T-cell acute lymphoblastic leukemia: a report of POG study 9296. Pediatric blood & cancer.

[6] Asselin BL, Devidas M, Wang C, Pullen J, Borowitz MJ, Hutchison R, Lipshultz SE, Camitta BM (2011). Effectiveness of high-dose methotrexate in T-cell lymphoblastic leukemia and advanced-stage lymphoblastic lymphoma: a randomized study by the Children's Oncology Group (POG 9404). Blood.

[7] McGowan CH, Russell P (1993). Human Wee1 kinase inhibits cell division by phosphorylating p34cdc2 exclusively on Tyr15. Embo J.

[8] Parker LL, Piwnica-Worms H (1992). Inactivation of the p34cdc2-cyclin B complex by the human WEE1 tyrosine kinase. Science.

[9] Porter CC, Kim J, Fosmire S, Gearheart CM, van Linden A, Baturin D, Zaberezhnyy V, Patel PR, Gao D, Tan AC, DeGregori J (2012). Integrated genomic analyses identify WEE1 as a critical mediator of cell fate and a novel therapeutic target in acute myeloid leukemia. Leukemia.

[10] Tibes R, Bogenberger JM, Chaudhuri L, Hagelstrom RT, Chow D, Buechel ME, Gonzales IM, Demuth T, Slack J, Mesa RA, Braggio E, Yin HH, Arora S, Azorsa DO (2012). RNAi screening of the kinome with cytarabine in leukemias. Blood.

[11] Van Linden AA, Baturin D, Ford JB, Fosmire SP, Gardner L, Korch C, Reigan P, Porter CC (2013). Inhibition of Wee1 sensitizes cancer cells to antimetabolite chemotherapeutics *in vitro* and *in vivo*, independent of p53 functionality. Mol Cancer Ther.

[12] Hirai H, Iwasawa Y, Okada M, Arai T, Nishibata T, Kobayashi M, Kimura T, Kaneko N, Ohtani J, Yamanaka K, Itadani H, Takahashi-Suzuki I, Fukasawa K, Oki H, Nambu T, Jiang J (2009). Small-molecule inhibition of Wee1 kinase by MK-1775 selectively sensitizes p53-deficient tumor cells to DNA-damaging agents. Mol Cancer Ther.

[13] Cook PJ, Ju BG, Telese F, Wang X, Glass CK, Rosenfeld MG (2009). Tyrosine dephosphorylation of H2AX modulates apoptosis and survival decisions. Nature.

[14] Chou TC, Talalay P (1984). Quantitative analysis of dose-effect relationships: the combined effects of multiple drugs or enzyme inhibitors. Adv Enzyme Regul.

[15] Prichard MN, Shipman C (1990). A three-dimensional model to analyze drug-drug interactions. Antiviral research.

[16] Dai Y, Grant S (2010). New insights into checkpoint kinase 1 in the DNA damage response signaling network. Clin Cancer Res.

[17] Ewald B, Sampath D, Plunkett W (2008). Nucleoside analogs: molecular mechanisms signaling cell death. Oncogene.

[18] Sampath D, Cortes J, Estrov Z, Du M, Shi Z, Andreeff M, Gandhi V, Plunkett W (2006). Pharmacodynamics of cytarabine alone and in combination with 7-hydroxystaurosporine (UCN-01) in AML blasts *in vitro* and during a clinical trial. Blood.

[19] Shi Z, Azuma A, Sampath D, Li YX, Huang P, Plunkett W (2001). S-Phase arrest by nucleoside analogues and abrogation of survival without cell cycle progression by 7-hydroxystaurosporine. Cancer Res.

[20] Karp JE, Thomas BM, Greer JM, Sorge C, Gore SD, Pratz KW, Smith BD, Flatten KS, Peterson K, Schneider P, Mackey K, Freshwater T, Levis MJ, McDevitt MA, Carraway HE, Gladstone DE (2012). Phase I and pharmacologic trial of cytosine arabinoside with the selective checkpoint 1 inhibitor Sch 900776 in refractory acute leukemias. Clin Cancer Res.

[21] Leijen S, Schellens JH, Shapiro G, Pavlick AC, Tibes R, Demuth T, Viscusi J, Cheng JD, Xu Y, Oza AM (2010). A phase I pharmacological and pharmacodynamic study of MK-1775, a Wee1 tyrosine kinase inhibitor, in monotherapy and combination with gemcitabine, cisplatin or carboplatin in patients with advanced solid tumors. J Clin Oncol.

[22] Caldwell JT, Edwards H, Buck SA, Ge Y, Taub JW (2014). Targeting the wee1 kinase for treatment of pediatric Down syndrome acute myeloid leukemia. Pediatr Blood Cancer.

[23] Weisberg E, Nonami A, Chen Z, Liu F, Zhang J, Sattler M, Nelson E, Cowens K, Christie AL, Mitsiades C, Wong KK, Liu Q, Gray N, Griffin JD (2014). Identification of Wee1 as a novel therapeutic target for mutant RAS-driven acute leukemia and other malignancies. Leukemia.

[24] Krajewska M, Heijink AM, Bisselink YJ, Seinstra RI, Sillje HH, de Vries EG, van Vugt MA (2013). Forced activation of Cdk1 via wee1 inhibition impairs homologous recombination. Oncogene.

[25] Christoph S, Schlegel J, Alvarez-Calderon F, Kim YM, Brandao LN, DeRyckere D, Graham DK (2013). Bioluminescence imaging of leukemia cell lines *in vitro* and in mouse xenografts: effects of monoclonal and polyclonal cell populations on intensity and kinetics of photon emission. Journal of hematology & oncology.

