# Supplementary material for: AZD1775 sensitizes T cell acute lymphoblastic leukemia cells to cytarabine by promoting apoptosis over DNA repair
Source: Oncotarget. 2015 Aug 10;6(29):28001–10. doi: 10.18632/oncotarget.4830 (PMC4695040; doi:10.18632/oncotarget.4830)
Supplement: Supplementary file 1 [file oncotarget-06-28001-s001.pdf]

SUPPLEMENTARY FIGURES AND TABLES

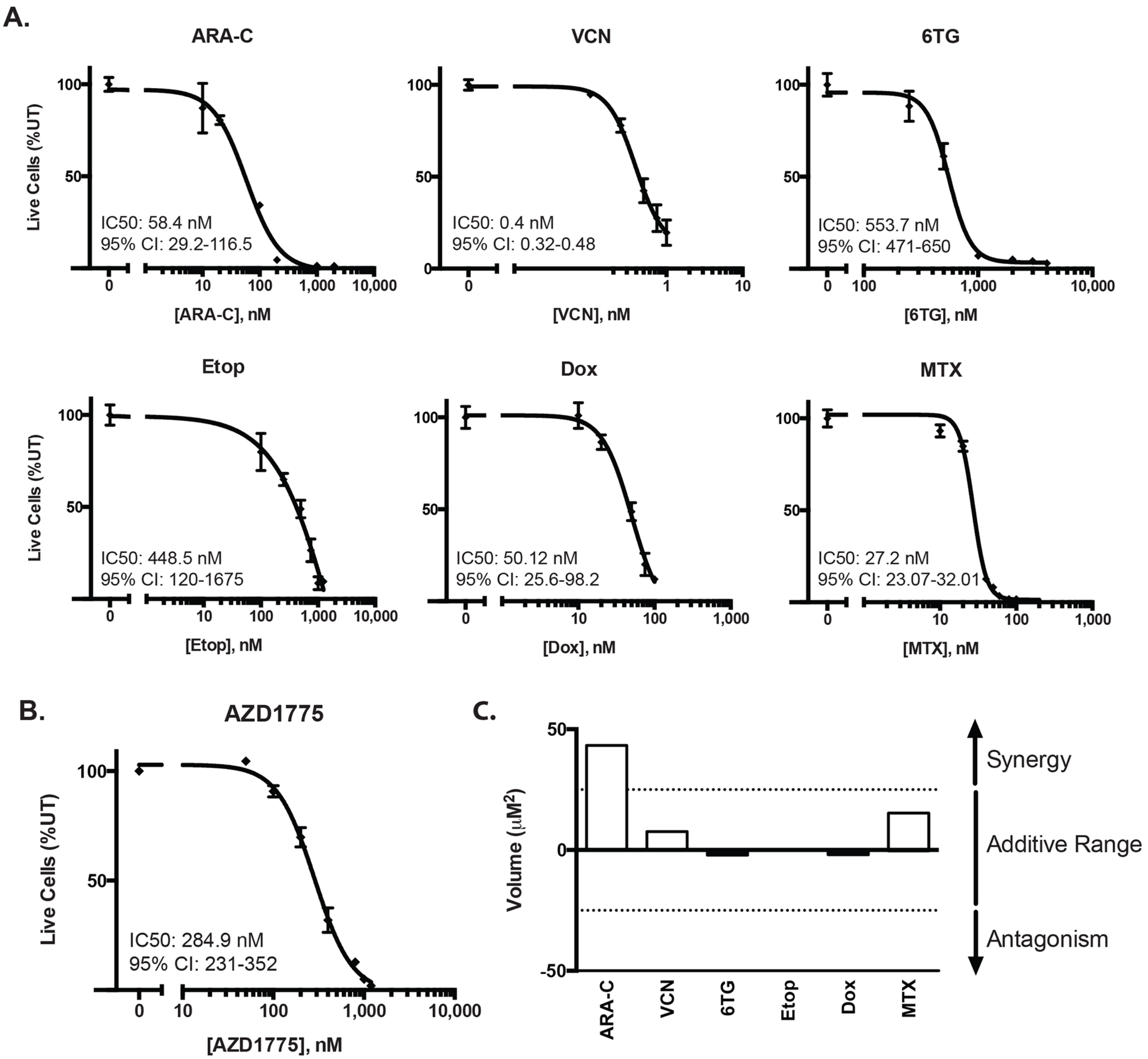

Supplementary Figure S1: AZD1775 is synergistic in combination with cytarabine. Jurkat cells were cultured for 72 hours at several doses of the indicated conventional chemotherapeutics **A.** or AZD1775 **B.** The number of live cells was determined and dose response curves were generated with GraphPad Prism using non-linear regression models, and the IC50 was calculated for each drug. **C.** Live cell numbers at 72 hours, relative to untreated controls, from at least 6 combinations of AZD1775 and the conventional chemotherapeutics were analyzed according to the Bliss independence model of drug interaction using MacSynergy II. Each experiment was performed at least 4 times, in triplicate. Volumes of synergy or antagonism calculated at the 95% confidence interval are depicted graphically. Values greater than 25 are considered as a significant synergistic interaction.

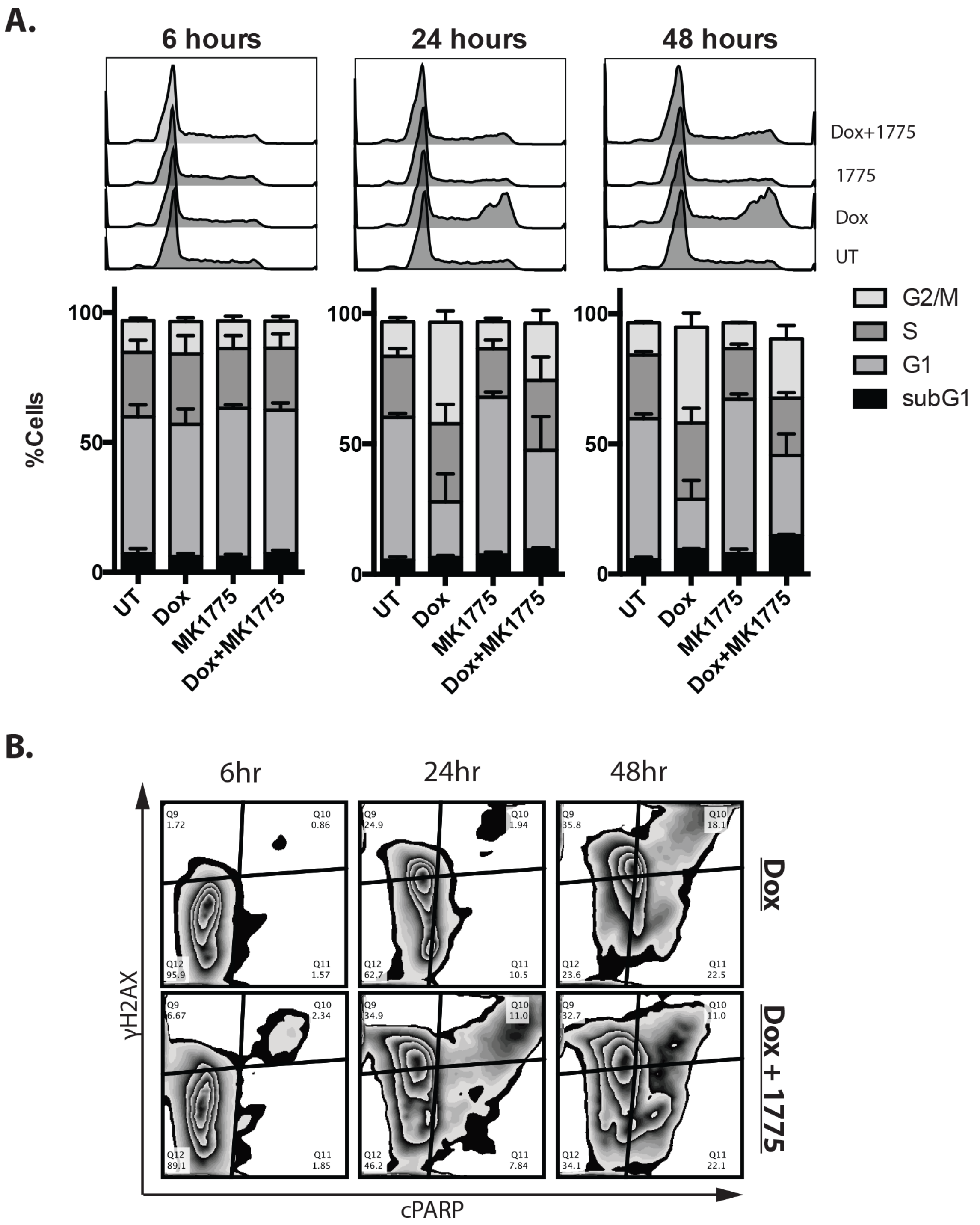

Supplementary Figure S2: AZD1775 promotes DNA damage and apoptosis with cytarabine but not doxorubicin. Jurkat cells were treated with doxorubicin, 50 nM, AZD1775 200 nM or in combination as indicated, and harvested at 6, 24 and 48 hours. **A.** Cells were fixed and stained with 7-AAD to assess cell cycle distribution by DNA content. **B.** Fixed and permeabolized cells were stained with fluorescently tagged antibodies directed against γH2AX and cleaved PARP (cPARP).

Supplementary Table S1: CI For experimental values

ARA-C (nM)	1775 (ARA-C) (nM)	Fa	CI
20	100	0.8491	0.592
20	200	0.9667	0.344
50	100	0.9675	0.328
50	200	0.978967	0.346

Supplementary Table S2: CI For experimental values

6TG (nM)	1775 (6TG) (nM)	Fa	CI
250	100	0.735635	1.048
250	200	0.840266	1.144
500	100	0.878752	1.091
500	200	0.860849	1.478

Supplementary Table S3: CI For experimental values

MTX (nM)	1775 (MTX) (nM)	Fa	CI
20	100	0.593115	1.29
20	200	0.711929	1.567
30	100	0.67592	1.48
30	200	0.78585	1.657

Supplementary Table S4: CI For experimental values

dox (nM)	1775 (dox) (nM)	Fa	CI
40	100	0.466963	1.379
40	200	0.680416	1.098
60	100	0.560393	1.481
60	200	0.727986	1.209

Supplementary Table S5: CI For experimental values

etop (nM)	1775 (etop) (nM)	Fa	CI
400	100	0.608009	0.975
400	200	0.789915	0.779
600	100	0.757836	0.878
600	200	0.805979	0.926

Supplementary Table S6: CI For experimental values

VCN (nM)	1775 (VCN) (nM)	Fa	CI
0.25	100	0.633067	1.156
0.25	200	0.895567	0.746
0.5	100	0.923033	0.763
0.5	200	0.979117	0.498
